# The effect of lipopolysaccharide on the expression level of immunomodulatory and immunostimulatory factors of human amniotic epithelial cells

**DOI:** 10.1186/s13104-018-3411-9

**Published:** 2018-05-29

**Authors:** Ramezan Ali Taheri, Hossein Motedayyen, Somayeh Ghotloo, Mohsen Masjedi, Nariman Mosaffa, Abbas Mirshafiey, Mahmood Saffari

**Affiliations:** 1 Nanobiotechnology Research Center, Baqiyatallah University of Medical Sciences, Tehran, Iran; 2 Department of Immunology, Faculty of Medicine, Isfahan University of Medical Sciences, Isfahan, Iran; 3 Department of Pathobiology, Faculty of Public Health, Tehran University of Medical Sciences, Tehran, Iran; 4 Department of Immunology, Faculty of Medicine, Shahid Beheshti University of Medical Sciences, Tehran, Iran; 5 Department of Microbiology and Laboratory Medicine, Kashan University of Medical Sciences, 5th kilometer of Ravand Road, Kashan, Iran

**Keywords:** Human amniotic epithelial cells, Toll-like receptors, Lipopolysaccharide, Immunomodulatory effects, Regenerative medicine

## Abstract

**Objective:**

Human amniotic epithelial cells (hAECs) are a novel source of stem cells and have immunomodulatory effects on both the innate and adoptive immune system. hAECs can differentiate into multiple cell lineages that make them a suitable cell source for regenerative medicine. These cells express multiple toll-like receptors (TLRs) and respond to various TLR ligands. This study aimed to evaluate the effect of lipopolysaccharide (LPS), a TLR4 ligand, on the level of immunomodulatory and immunostimulatory factors of hAECs.

**Results:**

Our results indicated that LPS had the ability to up-regulate the expression of prostaglandin E2 synthase and transforming growth factor-beta1 in hAECs. However, there was no change in the level of interleukin-1beta, interleukin-6 and interleukin-10 in hAECs when were stimulated with LPS. In addition, we observed tumor necrosis factor-alpha was only expressed at very low level in some of hAECs samples which its expression was independent of the effects of LPS.

**Electronic supplementary material:**

The online version of this article (10.1186/s13104-018-3411-9) contains supplementary material, which is available to authorized users.

## Introduction

The amnion membrane is the innermost layer of the fetal membranes and consists of epithelial cells and avascular stroma [1]. Human amniotic epithelial cells (hAECs) have unique properties that distinguish them from other human cell sources. hAECs express HLA-G which is an inhibitory ligand for B and T lymphocytes, natural killer (NK), and dendritic cells [2, 3] These cells have the ability to modulate immune responses through the secretion of soluble molecules and cell–cell contact [3]. hAECs utilize various immunosuppressive molecules such as TGF- β_1_, prostaglandin E2 (PGE2), and Fas ligand (Fas L) [4, 5]. These immunosuppressive agents play a crucial role in regulating immune response and preventing the development of autoimmune disorders through the inhibition of T cell proliferation, the shift of immune responses toward Th2-type responses and the reduction of pro-inflammatory responses [4].

In addition to immunomodulatory activities, these cells are proposed as a novel source of stem cells that differentiate into different cell types originating from three germ layers without any of the ethical concerns related to human stem cells [6]. hAECs express some of the functional toll-like receptors (TLRs) such as TLR2/TLR6, TLR4, and TLR5, which are important regulators of the innate immune system and recognize pathogens by conserved pathogen associated molecular patterns (PAMPs) [7]. TLR2/TLR6 and TLR5 stimulation on hAECs induce the production of IL-8 and IL-6 [7]. Previous studies indicated that the human placenta responds to different PAMPs by TLRs expression [8]. Activation of TLRs by PAMPs triggers intracellular signaling cascades resulting in the activation of nuclear factor-kappa B (NF-κB) and activation protein-1(AP-1), which are known as key transcription factors responsible for the expression of pro-inflammatory cytokines including IL-1, IL-6 and TNF-α. Pro-inflammatory cytokines stimulate the production of prostaglandins in the fetal membranes, thereby causing uterine contractions [7]. Therefore, engagement of TLRs by PAMP and, subsequently, the production of different factors of hAECs suggest that the fetal membranes play a pivotal role in the immune system.

Regarding the fact that the immunosuppressive and immunostimulatory effects of hAECs are mainly mediated by soluble molecules such as TGF-β1, PGE2, TNF-α and IL-6 [3–5], this study aimed to examine the effect of lipopolysaccharides (LPS), a ligand for TLR4, on the expression of soluble factors of hAECs such as TGF-β1, PGE2, IL-10, IL-1β, TNF-α and IL-6, which mediate the immunomodulatory and immunostimulatory effects of these cells.

## Main text

### Methods

#### hAECs isolation

Term placentas were obtained from 10 healthy pregnant women during uncomplicated elective cesarean deliveries. hAECs were isolated using a method described previously [9]. Briefly, the amnion membrane was manually peeled off from the chorion and washed several times with phosphate buffered saline (PBS). The amnion was then digested at 37 °C for 10 min with 0.05% EDTA/trypsin (Gibco, USA). The digestion of the amnion layer was followed twice at 37 °C for 30 min with 0.05% EDTA/trypsin. The isolated cells from the second and third digests were pooled and washed with ice-cold RPMI medium.

#### Assessment of the purity of hAECs by flow cytometry

To determine the purity of hAECs isolated from five amnion membranes, the cells (8 × 10^5^) were stained with FITC anti-human CD105, FITC anti-human CD90, and matched-isotype control IgG antibodies, as negative controls, at 4 °C for 25 min (Additional file 1: Table S1). Mesenchymal stem cells (MSCs) were used as positive control for anti-CD90 and anti-CD105 antibodies. Next, fixation and permeabilization of the cells were preformed for intracellular staining with Alexa Fluor^®^ 488 anti-human cytokeratin or matched-isotype control IgG antibodies (Additional file 1: Table S1) according to the manufacturere’s protocol (eBioscience, USA). The cells were then washed three times with cell staining buffer (Biolegend, USA) and the purity of the cells was analyzed using a FACSCalibur flow cytometer (Becton–Dickinson, CA).

#### hAECs culture

hAECs obtained from ten healthy pregnant women were cultured in 25 cm^2^ tissue culture flasks at a density of 2.5 × 10^5^ cells/cm^2^ in DMEM/F12 medium (Gibco, USA) supplemented with 10% FBS (Gibco, USA) and 1% penicillin/streptomycin (Sigma-Alderich, USA). LPS (1 mg, Sigma-Alderich, USA) was dissolved in 1 ml RPMI medium to yield a stock concentration of 1 mg/ml. After 24 h of incubation at 37 °C, one set of the cultured hAECs were stimulated with LPS (5 µg/ml) and incubated at 37 °C with 5% CO_2_. All assays were performed in duplicate and randomization was used while performing the experiment. After 6 h, adhered hAECs which were cultured in the presence or absence of LPS were dissociated by trypsin and washed twice with PBS for total RNA extraction.

#### RNA extraction, reverse transcription and quantitative polymerase chain reaction (qPCR)

For gene expression analysis, total RNAs from ten hAEC samples which were cultured in the presence or absence of LPS were extracted using the RNeasy Mini RNA isolation kit according to the manufacturer’s protocol (Qiagen, USA). The investigators were blinded to sample information. RNA yield was determined and the purity was assessed using a spectrophotometer (NanoDrop 8000 spectrophotometer, Thermo scientific, USA). Complementary deoxyribonucleic acid (cDNA) synthesis was done using RevertAid First Strand cDNA Synthesis Kit (Thermo scientific, USA) following the manufacturer’s instructions. q PCR assay was done using an ABI7700 machine (Applied Biosystems, Foster City, USA) and the SYBR^®^ Premix Ex Taq™ II Master Mix (Takara, Japan) according to the manufacturer’s instructions. Each reaction was initiated at 95 °C for 15 s, followed by 45 cycles of 95 °C for 5 s and 58 °C for 40 s. All analyses were performed in triplicate. Threshold cycles (Ct) and melting curves were generated automatically by the Applied Biosystems software. The expression levels of each sample were normalized to glyceraldehyde-3-phosphate dehydrogenase (GAPDH) as an endogenous control. The cycling parameters for GAPDH were similar to those used for the cytokines. The Relative Expression Software Tool 2009 (REST 2009) [10] was used to calculate the relative expression of the target genes, using the ratio of the Ct values and the PCR amplification efficiencies of the target genes and the GAPDH gene. REST 2009 uses randomization and bootstrapping methods to test the statistical significance of the gene expression ratios and calculate 95% confidence intervals for relative fold changes [11]. Primer sequences are shown in Additional file 1: Table S2.

#### Statistical analysis

The results are expressed as mean ± standard error (SE) and mean ± standard error of mean (SEM). REST 2009 was used for group-wise comparison and statistical analysis of relative expression results in real-time PCR. p < 0.05 was considered statistically significant.

### Results

#### The purity of hAECs

**Fig. 1:**
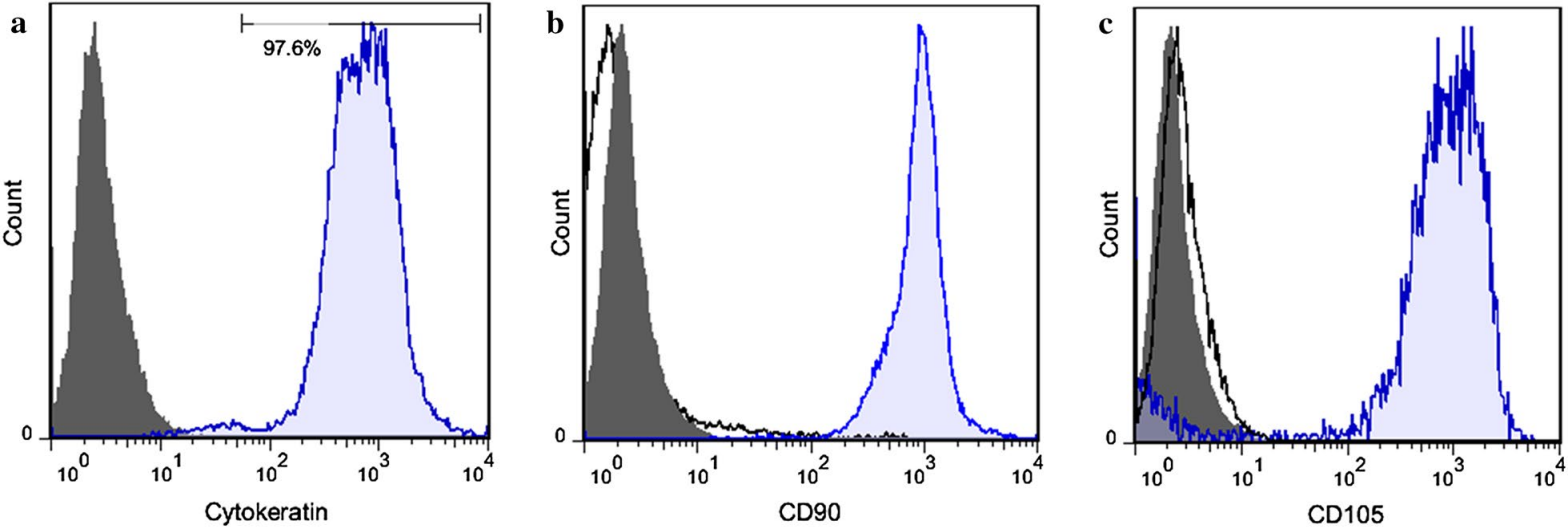
The purity of isolated hAECs isolated from the amnion membrane. **a** More than 97% of the isolated hAECs were positive for cytokeratin, an epithelial cell marker. **b**, **c** < 1% of the isolated cells were positive for MSC markers [CD90 (**b**) and CD105 (**c**)]. Blue shaded histogram: MSCs were stained with anti-CD90 (**b**) and anti-CD105 (**c**) antibodies as positive controls. Gray shaded histogram: hAECs were stained with matched-isotype control antibodies as negative controls (**a**–**c**). Black line: hAECs were stained with anti-cytokeratin (**a**), anti-CD90 (**b**) and anti-CD105 (**c**) antibodies. Data are representative of five independent experiments. All data show mean ± SEM

To assess the purity of hAEC, the percentage of cells which were positive for cytokeratin, CD90 and CD105 was measured by flow cytometry. Our results revealed that more than 97% of the isolated cells expressed cytokeratin and less than 1% of the cells were positive for CD90 and CD105 (Fig. 1a–c).

#### LPS effects on expression of immunosuppressive factors in hAECs

**Fig. 2:**
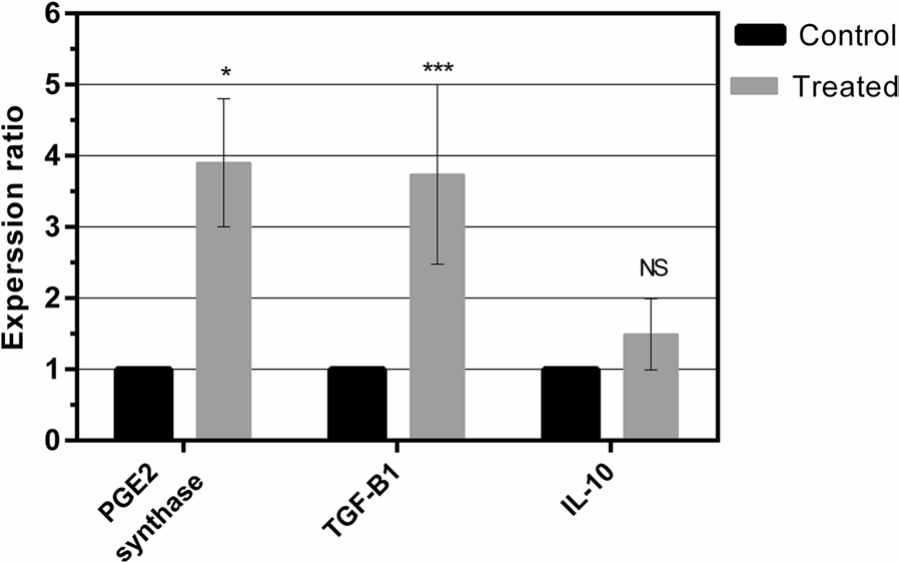
The expression level of immunosuppressive agents in hAECs. hAECs were cultured in the presence or absence of LPS for 6 h. The effects of LPS on the expression of PGE2 synthase, TGF-β1 and IL-10 in hAECs were evaluated by real-time PCR assay. All data are shown as mean ± SE. The depicted results are representative of ten independent experiments. Asterisk indicates that the difference in the expression levels are statistically significant. NS indicates that the difference in the expression levels is not statistically significant. *p < 0.05, ***p < 0.001

Since immunomodulatory effects of hAECs make them as a cell source for cellular therapy, LPS effects on expression of immunosuppressive mediators in hAECs was evaluated. Our data showed a significant increase in the expression level of PGE2 synthase, an enzyme which produces PGE2, and TGF-β1 in the sample group (hAECs treated with LPS) compared to the control group (hAECs cultured in the absence of LPS) (Fig. 2, p < 0.001–0.05). Although there was a numerical increase in the mean of IL-10 expression level in the sample group compared to the control group, LPS could not induce the IL-10 expression in the sample group (Fig. 2).

#### LPS effects on induction of pro-inflammatory cytokines in hAECs

**Fig. 3:**
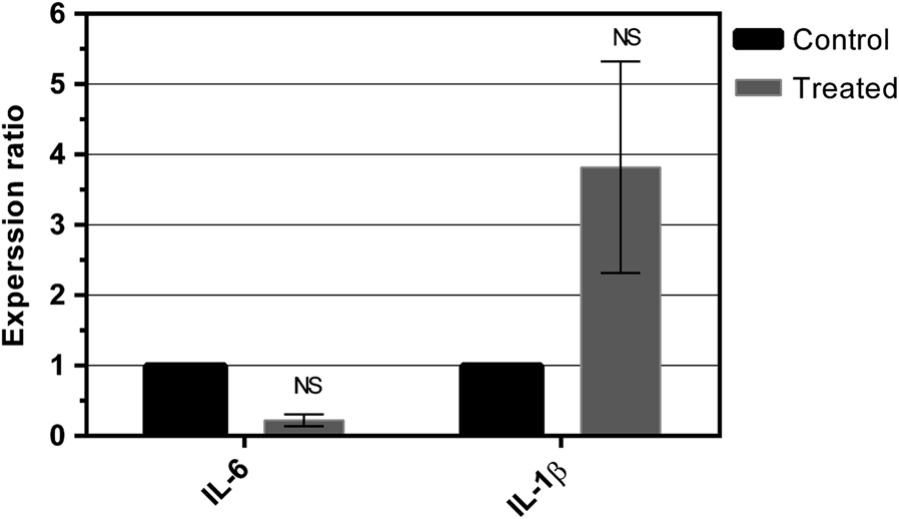
The expression level of pro-inflammatory cytokines in hAECs. hAECs were cultured in the presence or absence of LPS for 6 h. LPS effects on the expression of IL-1β and IL-6 cytokines in hAECs were evaluated by real-time PCR assay. Data are representative of ten independent experiments. All data show mean ± SE. NS indicates that the difference in the expression levels is not statistically significant

Regarding pro-inflammatory cytokines play an important role in the induction of immune response against the cells used with therapeutic purposes, we evaluated LPS effect on the expression of pro-inflammatory cytokines in hAECs. In spite of a numerical decrease in IL-6 level and an increase in IL-1β level in the sample group, the results of the study indicated that LPS did not influence the expression of IL-6 and IL-1β in hAECs (Fig. 3). Moreover, we found that TNF-α was expressed at very low level in hAECs and was only detectable in some samples such as sample 6, 7 and 10.

### Discussion

Immune-rejection and tumorigenic upon transplantation are two major problems which challenge the use of cell therapy for the treatment of different diseases [12, 13]. To date, multiple cell sources have been employed in regenerative medicine and the treatment of diseases with immune pathophysiology, but these treatments have not been uniformly successful and the evidence is insufficient to support the efficacy of these therapeutic approaches [12]. hAECs which are stem cells with the immunomodulatory effects may be considered as a potential candidate in the regenerative medicine and immunotherapy for inflammatory diseases. In addition, some unique characteristics of these cells make them an interesting source of cells for use as therapy in regenerative medicine. These cells express very low level of human leukocyte antigens (HLA) class I and are negative for HLA-class II and co-stimulatory molecules, which may potentially reduce the risk of immune-rejection after clinical applications [14]. hAECs do not express telomerase and thus cannot tumorigenic upon clinical use [3]. Therefore, the use of hAECs as a therapeutic approach has not challenged with the two major cell therapy issues, immunological rejection and tumor formation after transplantation.

TGF-β, IL-10, and PGE2 are known as powerful immunosuppressive molecules that play a pivotal role in regulation of the immune system [6, 15–19]. These factors impair the proliferation and differentiation of immune cells and inhibit the production of pro-inflammatory cytokines [4, 15, 19]. Several studies indicated that hAECs suppressed immune cells proliferation through TGF-β1 and PGE2 [3, 4]. Regarding the fact that hAECs express different TLRs and engagement of TLRs by PAMP influences hAECs function, the critical question was whether TLR-4 stimulation by LPS, as an important TLR for hAECs, affects the production of immunosuppressive and immunostimulatory mediators of hAECs.

In the current study, we observed that LPS had the ability to induce the expression of TGF-β_1_ and PGE2 synthase in hAECs but not for IL-10. For the first time, these results showed that the stimulation of hAECs with LPS may be considered as an approach for enhancing the immunoregulatory effects of these cells. However, the molecular mechanism involved in LPS effects on up-regulation of immunosuppressive molecules in hAECs is unknown. Therefore, this is a question that must be addressed in future studies. As mentioned before, no statistically significant increase of IL-10 level was observed in the LPS-treated hAECs. This finding may be explained in the context of very low expression of IL-10 in these cells, as some reports have shown that hAECs are unable to produce IL-10 [4, 14].

This study unexpectedly indicated that LPS did not affect the level of IL-1β and IL-6 in the hAECs. These results were in contrast with the known mechanism of TLR4 function, leading to activation of the NF-κB pathway and an increase in the production of pro-inflammatory cytokines [8]. However, there is a report that indicated the activation of TLR4 on hAECs by LPS influenced the viability of the cells, while cannot affect the production of pro-inflammatory cytokines by these cells [7]. In addition, it has been demonstrated that hAECs activation by TLR6/2, TLR 5 and TLR9 agonists produces IL-6 and IL-8, but not with LPS [7, 20]. In an effort to discover the effects of LPS on TNF-α expression, we found that there was an inconsistency in the expression of TNF-α in hAECs. In contrast with some studies indicating TNF-α expression by hAECs [5, 6], the results of this study demonstrated that the expression level of IFN-γ le was below the detection limit in hAECs, and the level was unchanged after stimulation with LPS. The IFN-γ level was only detectable in some of hAECs samples.

Taken together, the results of this study provide evidence to show that LPS may enhance the immunomodulatory effects of hAECs through up-regulating immunosuppressive factors and down-regulating pro-inflammatory cytokines in hAECs that may represent an advantageous cell source with potential applications for regenerative medicine. However, further studies are required to confirm the effects of LPS on immunomodulatory effects of hAECs and also explain the molecular mechanisms of LPS effects on hAECs.

## Limitation

The study was unable to determine whether LPS also affects the production of immunomodulatory and immunostimulatory factors of hAECs.

## Additional file

**Additional file 1: Table S1.** Antibodies used to determine the purity of hAECs by Flow cytometry. **Table S2.** Primer sequences used in SYBR-Green Based Real-Time PCR.

## Abbreviations

DMEM: Dulbecco’s modified eagle’s medium
RPMI: Roswell park memorial institute
PBS: phosphate-buffered saline
FBS: fetal bovine serum
hAECs: human amniotic epithelial cells
HBSS: Hanks’balanced salt solution
LPS: lipopolysaccharide
IL-1β: interleukin-1beta
TLR: toll-like receptor
PGE2: prostaglandin E2
TGF-β1: transforming growth factor-beta1
IL-6: interleukin-6
IL-10: interleukin-10
TNF-α: tumor necrosis factor-alpha
NF-κB: nuclear factor-kappa B
AP-1: activation protein 1
PAMPs: pathogen associated molecular patterns
