# Supplementary material for: The effect of lipopolysaccharide on the expression level of immunomodulatory and immunostimulatory factors of human amniotic epithelial cells
Source: BMC Res Notes. 2018 May 29;11:343. doi: 10.1186/s13104-018-3411-9 (PMC5975661; doi:10.1186/s13104-018-3411-9)
Supplement: Supplementary file 1 — Additional file 1: Table S1. Antibodies used to determine the purity of hAECs by Flow cytometry. Table S2. Primer sequences used in SYBR-Green Based Real-Time PCR. [file 13104_2018_3411_MOESM1_ESM.docx]

**Additional file 1**

**Table S1.** Antibodies used to determine the purity of hAECs by Flow cytometry

| **Fluorochrome/Antibody** | **Isotype control** | **Catalog number** | **Working volume** | **Company (All from USA)** |
| --- | --- | --- | --- | --- |
| Alexa Fluor® 488 anti-Cytokeratin (pan reactive) | Mouse IgG1, κ | 628608 | 5µl/test | Biolegend |
| FITC anti-human CD105 | Mouse IgG1, κ | 323203 | 5µl/test | Biolegend |
| FITC anti-human CD90 | Mouse IgG1, κ | 328107 | 5µl/test | Biolegend |

**Table S2.** Primer Sequences used in SYBR-Green Based Real-Time PCR

| **Gene** | **Forward Primer (5ʹ to 3ʹ)** | **Reverse Primer (5ʹ to 3ʹ)** |
| --- | --- | --- |
| PGE synthase | CCATCATCACGGGCCAAGT | GTCTCCATGTCGTTCCGGTG |
| TGF-β1 | TCCTGGCGATACCTCAGCAA | AGTGAACCCGTTGATGTCCA |
| IL-10 | CGAGATGCCTTCAGCAGAGT | CGCCTTGATGTCTGGGTCTT |
| IL-1β | GCACGATGCACCTGTACGAT | TGGAGAACACCACTTGTTGC |
| TNF-α | GCTGCACTTTGGAGTGATCG | GGGTTTGCTACAACATGGGC |
| IL-6 | CTTCTCCACAAGCGCCTTCG | CTGAGATGCCGTCGAGGATG |
| GAPDH | CTCTGGTAAAGTGGATATTG | GGTGGAATCATATTGGAACA |
